# The Expression, Functions and Mechanisms of Circular RNAs in Gynecological Cancers

**DOI:** 10.3390/cancers12061472

**Published:** 2020-06-04

**Authors:** Peixin Dong, Daozhi Xu, Ying Xiong, Junming Yue, Kei Ihira, Yosuke Konno, Hidemichi Watari

**Affiliations:** 1 Department of Obstetrics and Gynecology, Hokkaido University School of Medicine, Hokkaido University, Sapporo 060-8638, Japan; xudaozhi87@yahoo.co.jp (D.X.); ihey0610@huhp.hokudai.ac.jp (K.I.); konsuke013@gmail.com (Y.K.); 2 Department of Gynecology, State Key Laboratory of Oncology in South China, Sun Yat-sen University Cancer Center, Guangzhou 510060, China; tdken999@163.com; 3 Department of Pathology and Laboratory Medicine, University of Tennessee Health Science Center, Memphis, TN 38163, USA; jyue@uthsc.edu; 4 Center for Cancer Research, University of Tennessee Health Science Center, Memphis, TN 38163, USA

**Keywords:** circular RNA, gynecological cancer, biomarker, non-coding RNA, cancer diagnosis, cancer treatment

## Abstract

Circular RNAs (circRNAs) are covalently closed, endogenous non-coding RNAs and certain circRNAs are linked to human tumors. Owing to their circular form, circRNAs are protected from degradation by exonucleases, and therefore, they are more stable than linear RNAs. Many circRNAs have been shown to sponge microRNAs, interact with RNA-binding proteins, regulate gene transcription, and be translated into proteins. Mounting evidence suggests that circRNAs are dysregulated in cancer tissues and can mediate various signaling pathways, thus affecting tumorigenesis, metastasis, and remodeling of the tumor microenvironment. First, we review the characteristics, biogenesis, and biological functions of circRNAs, and describe various mechanistic models of circRNAs. Then, we provide a systematic overview of the functional roles of circRNAs in gynecological cancers. Finally, we describe the potential future applications of circRNAs as biomarkers for prognostic stratification and as therapeutic targets in gynecological cancers. Although the function of most circRNAs remains elusive, some individual circRNAs have biologically relevant functions in cervical cancer, ovarian cancer, and endometrial cancer. Certain circRNAs have the potential to serve as biomarkers and therapeutic targets in gynecological cancers.

## 1. Introduction

The Encyclopedia of DNA Elements (ENCODE) project consortium reported that only 2% of the transcribed RNAs are translated into proteins, whereas the majority of other transcripts have no apparent coding potential [1]. Increasing evidence suggests that a significant part of such non-protein coding RNAs (ncRNAs) participate in complex regulatory networks composed of other nucleic acids or proteins [2,3]. The complexity of these networks allows ncRNAs to regulate the expression of a broad spectrum of genes [2,3].

Generally, ncRNAs can be separated into small non-coding RNAs (small ncRNAs), which are shorter than 50 nt, medium non-coding RNAs, which are between 50–200 nt, and long non-coding RNAs (lncRNAs), which are longer than 200 nt [4]. MicroRNAs (miRNAs) are the most studied small ncRNAs and they typically bind to the 3′-untranslated regions (3′-UTR) of protein-coding mRNA by imperfect sequence-specific recognition to either degrade it or repress its translation [5]. LncRNAs can be further subdivided into various categories, including linear RNAs and circular RNAs (circRNAs) [4]. Linear lncRNAs are involved in a broad range of biological processes and are key regulators of cancer metastasis [6,7].

CircRNAs were first discovered in the 1970s [8,9] and were initially thought to be a non-functional by-product of aberrant splicing in cells [10]. However, recent advances in sequencing technologies have revealed that large numbers of circRNAs are broadly expressed in a wide range of mammalian tissue [11]. Over 30,000 circRNAs have already been found in human tissues [12]. Due to their circular form and lack of free ends, circRNAs are resistant to RNA degradation by an exonuclease and have greater stability than linear RNAs [13]. Although the functions of most circRNAs are still unknown, at least some individual circRNAs are novel players in normal physiological and pathological conditions [14,15].

In this review, we discuss the characteristics, biogenesis, biological functions, and working mechanisms of circRNAs, with a particular emphasis on important findings concerning gynecological cancers.

## 2. General Characteristics of circRNAs

CircRNAs are covalently-closed RNA molecules, and they are produced by a non-canonical splicing process called “back-splicing”, which takes place in the nucleus [16]. Unlike typical linear RNAs, the 5′ cap and 3′ polyadenylation tail (poly-(A) tail) are absent in circRNAs. Therefore, circRNAs are not easily degraded by an exonuclease and are considerably more stable than linear RNAs. The average half-life of circRNAs in mammary cells is at least 2.5–4.8 times longer than the median half-life of their linear counterparts [13,17]. Following biogenesis, the majority of circRNAs, with the exception of intron-containing circRNAs that mainly exist in the nucleus, are exported to the cytoplasm in a size-dependent manner [18].

On average, circRNAs contain 1–5 exons and are 500 nt long [16]. The expression of a circular RNA does not often correlate with the expression of its cognate linear mRNA [16]. Of note, some circRNAs are expressed at a much lower or higher level than their linear counterparts [16]. Many circRNAs are highly conserved across mammals [17]. circRNAs exhibit dynamic expression levels during development and are expressed in a tissue-specific manner [19,20,21].

## 3. Mechanisms of circRNA Formation and Degradation

Although the mechanism of circRNA formation is still unclear, the majority of circRNAs are considered to originate from exons in the coding region of a gene, and the rest originate from the 5′- or 3′-UTRs, introns and intergenic regions, as well as from antisense RNAs [15]. According to their structural domains, circRNAs can be divided into four categories: exonic circRNA (ecircRNA), circular intronic RNA (ciRNA), exonic-intronic circRNA (EIciRNA) and intergenic circRNA [22]. EcircRNAs appear to be the most abundant circRNA type, accounting for over 80% of known circRNAs [23]. There are currently three hypothetical models to explain the formation of ecircRNAs: lariat-driven circularization, intron-pairing-driven circularization, and RNA-binding protein-mediated circularization [24,25]. The generation of ecircRNAs is a result of pre-mRNA splicing when the 3′ splice donor attaches to the 5′ splice acceptor [24,25]. Researchers have also demonstrated that RNA-binding proteins can serve as activators or inhibitors in the formation of circRNAs [24,25]. For instance, Quaking preferentially binds to a particular sequence motif on a linear RNA and bridges the two bracketing introns together, thus facilitating the formation of circRNAs in human mammary epithelial cells [26].

Compared to the mechanisms of circRNA formation, our understanding of circRNA degradation is limited. Some miRNAs can direct Ago2 to degrade circRNAs in human lung cancer cells and HEK-293 cells [27,28]. A previous study reported that an endonuclease, RNase P/MRP, cuts N6-methyladenosine (m^6^A)-containing circRNA in a sequence-dependent manner [29]. Upon viral infection, the activation of the endonuclease RNase L is responsible for circRNAs degradation [30]. A good number of circRNAs in human tumor cells are predicted to be highly structured, and their decay is regulated by UPF1 and G3BP1, two RNA-binding proteins [31]. In addition, human tumor cells can remove cytoplasmic circRNAs by releasing cargo-bearing extracellular vesicles, such as microvesicles and exosomes [32,33].

## 4. Biological Functions and Mechanistic Models of circRNAs in Tumors

**Figure 1:**
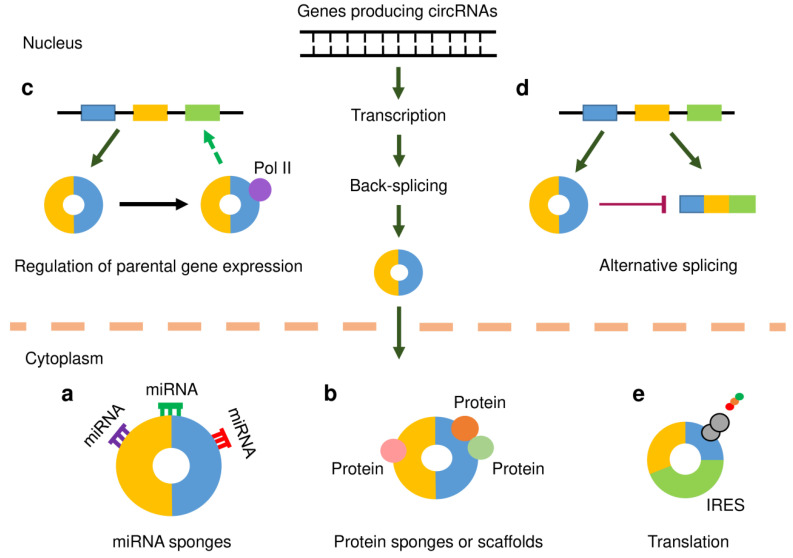
Functional mechanisms of circular RNAs (circRNAs). CircRNAs, like mRNAs, are transcribed from protein-coding genes by RNA polymerase II (Pol II). However, they are spliced in a non-canonical splicing process known as back-splicing. Following biogenesis, the majority of circRNAs are exported to the cytoplasm in a size-dependent manner. (**a**) CircRNAs may act as microRNA (miRNA) sponges by binding to miRNAs and inhibiting their functions, thereby releasing downstream target genes from miRNA-mediated repression. (**b**) CircRNAs that harbor binding sites for RNA-binding proteins (RBPs) may function as protein sponges and thus regulate gene expression. In addition, circRNAs may work as scaffolds in the assembly of protein complexes. (**c**) CircRNAs may associate with Pol II to enhance the transcription of their parental genes. (**d**) CircRNAs may compete with their linear counterparts against canonical pre-mRNA splicing, thus suppressing the expression of their parent genes. (**e**) CircRNAs that contain internal ribosomal entry site (IRES) elements and open reading frame may be translated into protein or polypeptide.

In human tumor cells, circRNAs are believed to serve as miRNA (or protein) sponges, scaffolds for proteins, regulators of transcription or splicing, and a few circRNAs can be translated into peptides or proteins under certain conditions (Figure 1). Some circRNAs can regulate cancer metastasis and affect the tumor microenvironment.

### 4.1. MiRNA Sponge

One of the most frequently studied functions of circRNA is its ability to act as a miRNA sponge. Recent observations have found that some lncRNAs act as competing endogenous RNAs (ceRNAs), which regulate other RNA transcripts by competing for shared miRNAs [25] (Figure 1a). CircRNAs that harbor multiple binding sites for miRNAs can function as efficient miRNA sponges to indirectly regulate the expression of protein-coding genes [34]. For example, circHIPK3 modulates tumor cell growth by silencing nine miRNAs [23]. Despite the consistency of these findings, there are a few examples that challenge this ceRNA hypothesis for circRNAs [35,36]. For example, bioinformatics analysis has indicated that miRNA-binding sites are generally not enriched in circRNAs any more than could be expected by chance, with the exception of the reported circRNA, ciRS-7 [35]. In another study, the circRNAs downloaded from the circBase database were screened for Ago2-bound regions [36]. As a result, 58,063 circRNAs were found to own Ago2-bound regions. Six circRNAs were selected, and the potential ability of these circRNAs to interact with miRNAs was explored using RNA-binding protein immunoprecipitation-PCR experiments. The results revealed that these six circRNAs did not exhibit strong potential to serve as miRNA sponges [36].

### 4.2. Protein Sponge and Scaffold

RNA-binding proteins are a class of proteins that bind RNAs to regulate the metabolic processing of RNAs by mediating their maturation, transport, localization, translation and turnover [37] (Figure 1b). CircRNAs that contain multiple complementary binding sites to one or multiple proteins might function as protein sponges. Several circRNAs were suggested to sequester HuR, an extensively studied RNA-binding protein, which consequently affects its action on target gene regulation [38,39,40]. The interactions between circRNAs and RNA-binding proteins have been shown to influence cancer progression [41]. In addition, circRNAs were observed to act as dynamic scaffolds that bring different proteins together to form a complex [41]. In mouse fibroblast cells, circ-Foxo3 can interact with both p21 and CDK2 to form a ternary complex that results in the inhibition of cell cycle progression [42].

### 4.3. Transcription and Splicing

Unlike ecircRNAs, which are located in the cytoplasm, ciRNAs and EIciRNAs are primarily enriched in the nucleus. Previous studies have shown that a class of EIciRNAs interact with RNA polymerase II (Pol II) and accumulate at transcription sites, thereby promoting the transcription of their parental genes [43,44] (Figure 1c). Moreover, certain circRNA can compete with their linear counterparts against canonical pre-mRNA splicing, thus suppressing the expression of their parent genes [45,46] (Figure 1d).

### 4.4. Translation of circRNAs

CircRNAs were initially regarded as ncRNAs that lack protein-coding abilities, owing to the absence of a 5′ cap structure and poly-(A) tail. However, subsequent studies have revealed that some circRNAs carry open reading frames, and can be translated into peptides or proteins [47,48] (Figure 1e). The presence of internal ribosomal entry sites (IRES) was shown to drive circRNA translation through a cap-independent mechanism [49]. N6-methyladenosine (m6A) refers to the methylation that occurs in the mRNAs, and the presence of m6A on circRNA has the potential to initiate cap-independent translation [50]. The peptides or proteins encoded by circRNAs have critical functions in mediating cancer development [51,52]. Therefore, it seems necessary to determine whether the proteins originating from circRNA translation are functional in gynecological cancers.

## 5. Methods for Detecting and Characterizing circRNAs

The detailed description of bioinformatic tools, laboratory techniques and general workflow used for circRNA research has been reviewed elsewhere [53,54,55,56,57].

Previous studies have demonstrated that tumor-adjacent tissues have similar molecular changes to those found in the tumor tissues or present a unique intermediate state between healthy and tumor tissues [58,59,60]. One example is miR-29c, which is significantly upregulated in tumor-adjacent tissues when compared with gastric cancer tissues as well as non-cancerous tissues [60]. Most of the previous studies that analyze circRNA global expression in human cancer tissues have only used matched tumor-adjacent samples as normal control [61,62]. The results indicate that the expression of circRNAs in cancer tissues is frequently downregulated when compared with the matched tumor-adjacent tissues [61,62]. However, some circRNAs are actually overexpressed in tumor-adjacent tissues in comparison to cancer samples and normal samples without cancer [63], suggesting that the use of tumor-adjacent tissues for comparison purposes may lead to experimental biases regarding the true circRNA expression profile associated with tumors [63].

The functional characterization of circRNAs is commonly conducted by following two types of strategies: RNA interference (RNAi)-induced circRNA silencing and gain-of-function studies using circRNA expression plasmids. One of the challenges for circRNA overexpression is that the vectors used for overexpressing circRNA might generate many undesired transcripts, such as linear RNAs or concatenated and/or intertwined circRNAs [64,65]. Therefore, it might be difficult to determine if a specific circRNA has an impact on a particular phenotype in tumor cells. Although recently developed expression vectors were believed to efficiently drive circRNA formation, but not linear RNAs [66], the combination of RNAi-based gene silencing with vector-mediated overexpression approaches may present more opportunities to fully understand the exact functions of circRNAs in tumor cells.

Recently, the novel CRISPR/Cas13-based approaches using guide RNAs, which target the circRNA-specific back-splicing junction sites, have been used to achieve precise and robust circRNA silencing without disturbing their linear cognate mRNAs [67,68]. These studies suggest that the application of CRISPR/Cas-based circRNA engineering systems may be a promising method for performing functional studies of circRNA in human cancers.

## 6. Evidence Acquisition

**Figure 2:**
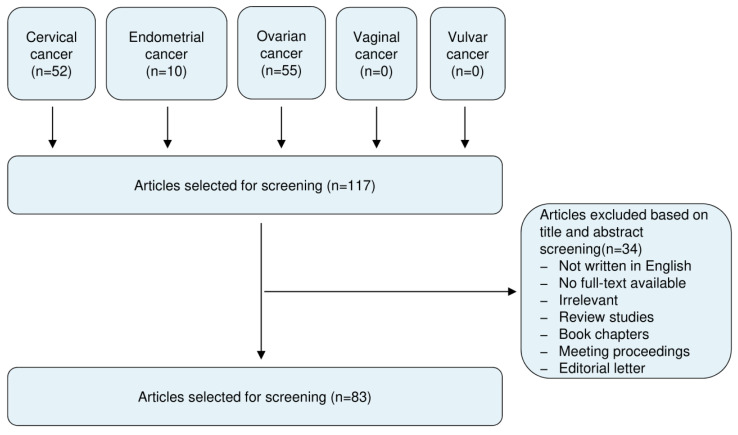
Summary of literature search, screening and selection.

This review included literature from the PubMed and Google Scholar databases published up to 20 February 2020, using the following keywords: circular RNA, circRNA, cervical cancer, endometrial cancer, ovarian cancer, vaginal cancer and vulvar cancer. All studies recognized were assessed for relevance by two authors by checking the title and abstract. After excluding the irrelevant articles, studies without access to the full-text of the publication, case reports, letters, expert opinions, meeting proceedings, review articles and non-English articles, our screening identified 83 articles that addressed the expression, roles and mechanisms of circRNAs in cervical cancer (CC), ovarian cancer (OC) and endometrial cancer (EC). These selected articles were evaluated independently by two authors. A flow diagram of the study selection process was shown in Figure 2.

## 7. The Expression, Functions and Mechanisms of circRNAs in Gynecological Cancers

### 7.1. CircRNAs in CC

**Figure 3:**
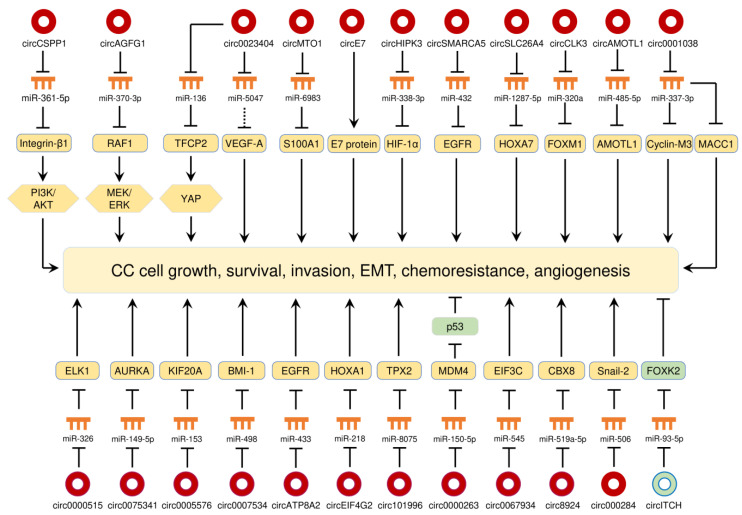
The circRNA-miRNA-mRNA regulatory networks and an HPV E7 oncoprotein-encoding circRNA play crucial roles in controlling CC progression, angiogenesis and chemoresistance. Red circles: oncogenic circRNAs; green circles: tumor-suppressive circRNAs.

CC is the fourth most common female malignancy in the world and kills more than 300,000 deaths worldwide each year [69]. High-risk subtypes (16 and 18) of human papillomavirus (HPV) are well established as the causative agents responsible for CC [69]. Massive studies have shown that a large number of circRNAs were differentially expressed in CC tissues compared with adjacent normal cervical tissues, and play oncogenic or tumor-suppressive roles in the initiation and progression of CC [70,71,72,73,74,75,76,77,78,79,80,81,82,83,84,85,86,87,88,89,90,91,92,93,94,95,96,97,98,99,100,101,102,103,104,105]. All of the circRNAs that are abnormally expressed in CC are summarized in Table 1, and their functions in CC are shown in Figure 3. As demonstrated by many studies, circRNAs function as miRNA sponges to regulate different aspects of CC progression through regulating multiple signaling pathways (Figure 3). These studies demonstrate that the circRNA-miRNA-mRNA regulatory networks and an HPV E7 oncoprotein-encoding circRNA play crucial roles in controlling CC tumorigenesis and progression.

#### 7.1.1. CircRNAs as Oncogenes in CC

##### CircRNAs Promote CC Growth, Survival, Invasion, EMT and Chemoresistance

Diverse circRNAs (such as circEIF4G2, circ0007534, circ0005576, circ0075341, circ0000515, circAMOTL1, and circSLC26A4) were previously described to promote the tumorigenesis of CC by increasing the respective downstream targets mRNAs, including HOXA1, BMI-1, KIF20A, AURKA, ELK1, AMOTL1 and HOXA7 via sponging miRNAs [84,90,91,94,95,97,101]. Additionally, circRNAs (such as circ0018289, circ0000285 and circMYBL2,) were found to be overexpressed in CC tissues, and their upregulation enhanced CC cell invasion and metastasis [76,92,98].

The PI3K/AKT signaling pathway controls multiple cellular processes including proliferation, growth, invasion and chemoresistance, and it is one of the most frequently dysregulated pathways in human cancers, including CC [106,107]. Integrin β1 can promote cancer initiation and progression through the activation of multiple downstream pathways, including the PI3K/AKT signaling [108]. By sponging miR-361-5p, circCSPP1 stimulates the expression of integrin β1, allowing downstream activation of the PI3K/AKT pathway, which contributes to CC cell proliferation and migration [105]. The RAF/MEK/ERK cascade plays a critical role in the development and progression of cancer [109]. A recent study suggested that circAGFG1 was upregulated in CC cell lines compared with normal cervical cells, and it could induce CC cell proliferation and migration by elevating RAF1 expression and activating the MEK/ERK pathway, via sponging miR-370-3p (a suppressor of RAF1) [93]. Epithelial-mesenchymal transition (EMT) is a biological process in which epithelial cells acquire mesenchymal phenotypes, thus conferring the malignant properties to cancer cells including invasive behaviors, cancer stem cell properties, and greater resistance to cancer treatment [110]. Experimental studies have revealed that circRNAs affect the EMT program in human cancers (including CC) through different mechanisms [77,80,89,99,100,104,111]. For instance, circ000284 indirectly increases Snail-2 expression to promote EMT and invasion of CC cells through sponging miR-506 [77]. CircCLK3 induces the levels of FOXM1 via sponging miR-320a, and overexpression of FOXM1 could trigger EMT-like changes, including the acquisition of Vimentin and N-cadherin expression and reduction of E-cadherin expression [99]. Also, the upregulation of circ0067934, circ0000745, ciRS-7 and circHIPK3 in CC cells was shown to increase the expression of mesenchymal markers and decrease the expression of epithelial markers [80,89,100,104]. Inactivation of p53 in human tumors occurs through missense mutations in the DNA-binding domain of the p53 protein, and can also result from the amplification/overexpression of its specific inhibitors (such as MDM2 and MDM4) [112]. Circ0000263 can increase the expression of MDM4 by sponging miR-150-5p, thus causing degradation of p53 in CC cells [81].

Circ0023404 enhanced chemoresistance of CC cells to cisplatin by regulating autophagy signaling [86]. Moreover, another circRNA, circMTO1, was shown to upregulate S100A1 expression through sponging miR-6893, thereby promoting tumorigenesis and chemoresistance in CC [88].

##### CircRNA Encodes HPV E7 Oncoprotein

The oncoproteins E6 and E7 encoded by HPV16 and 18 contribute to the neoplastic transformation of squamous cervical epithelium [113]. The E6 protein from high-risk HPVs binds the p53 tumor suppressor protein for degradation [114], whereas the E7 protein interacts with the retinoblastoma protein and inactivates this cellular protein [115]. A recent report showed that oncogenic HPV16 generates a 472nt circular RNA called circE7, which contains the entire E7 open reading frame [87]. In this study, circE7 was shown to be translated to the E7 oncoprotein, and knockdown of circE7 in CC-derived CaSki cells reduced E7 protein levels and suppresses tumor growth and malignancy both in vitro and in vivo [87]. These results suggest that circE7 appears to be essential for E7 protein expression and the transformation of CaSki cells.

##### CircRNAs as Tumor Suppressors in CC

Recent data suggest that circSMARCA5 was downregulated in CC tissues compared with adjacent normal tissues, and overexpression of circSMARCA5 suppresses the proliferation and invasion of HeLa cells by sponging miR-620 [82]. However, controversial findings have been reported in another study showing that circSMARCA5 was expressed at higher levels in CC tissues, and knockdown of circSMARCA5 suppressed cellular growth and invasion [102]. The oncogenic functions of circSMARCA5 were mediated through a miR-432/EGFR axis [102]. Besides, the expression of circITCH was significantly decreased in CC tissues compared with the adjacent normal tissues [103]. CircITCH acts as an endogenous miR-93-5p sponge to induce the levels of FOXK2 in CC cells, leading to the suppression of the malignant behaviors [103].

### 7.2. CircRNAs in OC

**Figure 4:**
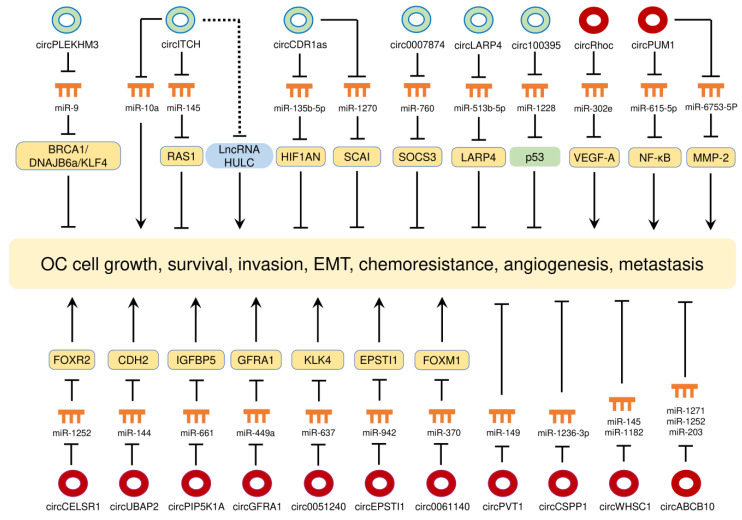
The circRNA-miRNA-mRNA regulatory networks play essential roles in mediating ovarian cancer progression, angiogenesis and chemoresistance. Red circles: oncogenic circRNAs; green circles: tumor-suppressive circRNAs.

Expression profiling studies and bioinformatics analysis have revealed significant changes in circRNA expression between OC tissues and normal ovarian tissues [116,117], between miliary-type OC and non-miliary OC [118], between serum samples from OC patients and those samples from healthy controls [119], between primary and metastatic sites of OC [120], and between OC cells and non-malignant human ovarian surface epithelial cells [62]. The expression and mechanisms of circRNAs in OC are presented in Table 2 and their biological roles are shown in Figure 4.

#### 7.2.1. CircRNAs as Oncogenes in OC

##### CircRNAs Promote CC Growth, Survival, Invasion, EMT, Chemoresistance and Metastasis

Several circRNAs (such as circEPSTI1, circGFRA1, circ0051240, circPIP5K1A, circPVT1 and circABCB10) have been found to increase the proliferative and invasive capacities of OC cells by acting as miRNA sponges [121,122,123,124,125,126]. Knockdown of circSMAD7 by short-hairpin RNA (shRNA) was associated with decreased cell proliferation, migration and invasion and with increased expression of KLF6 in CC cells [127]. Silencing of circANKRD12 reduced OC cell proliferation, probably through downregulating the expression of cyclin D1 [128].

CircCSPP1 was shown to be highly expressed in OC tissues and borderline tumor tissues when compared with normal ovarian tissues [129]. Further investigations suggest that circCSPP1 sponged miR-1236-p to increase the expression of ZEB1, leading to increased cell invasion and EMT phenotypes [129]. Circ0061140, which was upregulated in OC cell lines relative to normal ovarian epithelial cells, was reported to stimulate OC cell proliferation, invasion and EMT properties, probably via binding to miR-370 [130]. Overexpression of FOXM1, a target of miR-370, rescued the EMT properties that were reduced by either circ0061140 silencing or miR-370 overexpression [130]. By sponging miR-144, circUBAP2 promoted the expression of N-cadherin in OC cells [131]. CircWHSC1 was increasingly expressed in OC tissues compared with normal ovarian tissues, and there was a tendency towards increased levels of circWHSC1 expression in poorly- and moderately-differentiated OCs compared with well-differentiated OCs [132]. By interacting with miR-145/miR-1182, circWHSC1 was shown to elevate the levels of two EMT-inducer genes (MUC1/hTERT) in OC cells [132,133,134]. Inhibition of a circRNA VPS13C-has-circ-001567 by shRNA attenuated cellular invasion, resulting in the downregulation of N-cadherin and upregulation of E-cadherin [135].

CircCELSR1 acts as a sponge for the tumor suppressor miR-1252, and suppression of miR-1252, in turn, contributes to paclitaxel resistance in OC cells, possibly by inducing the expression of its target gene *FOXR2* [136].

CircPUM1 was found to be highly expressed in OC tissues compared with normal ovarian tissues, and higher circPUM1 expression was positively correlated with advanced FIGO stage [137]. Studies in both OC cell lines and mouse OC models suggest that circPUM1 promoted cell invasiveness and growth by sponging miR-615-5p and miR-6753-5p to upregulate NF-κB and MMP2 expression [137]. Interestingly, circPUM1 was present in OC cell-derived extracellular vesicles, and exosomal circPUM1 facilitated tumor peritoneal dissemination in vivo [137].

##### CircRNAs Promotes Angiogenesis in OC

Angiogenesis is influenced by the microenvironment and tightly regulated by a balance between pro- and anti-angiogenic factors [138]. Vascular endothelial growth factor (VEGF-A) has a prominent role in tumor angiogenesis [139]. CircRhoC, a circRNA derived from the *RhoC* mRNA, was overexpressed in OC tissues relative to normal ovarian tissues, and higher circRhoC expression was detected in advanced-stage tumors compared with early-stage tumors [140]. Suppression of circRhoC with shRNA significantly attenuated cell migration and invasion in vitro, and inhibited the intraperitoneal dissemination of OC cells in vivo [140]. By performing mechanistic analysis, the same study indicated that circRhoC can serve as a sponge for miR-302e to upregulate the expression of VEGF-A, which potentially promotes OC angiogenesis and metastasis [140].

#### 7.2.2. CircRNAs as Tumor Suppressors in OC

Some circRNAs exert their tumor-suppressive roles in OC cells [141,142,143,144,145,146,147,148,149,150] (Table 2 and Figure 4). According to these reports, the “miRNA” sponge seems to be the most common mechanism by which circRNAs regulate the malignant properties of OC cells. Interestingly, one study proposed a different potential mechanism whereby circITCH might inhibit the proliferation of OC cells by downregulating the levels of lncRNA HULC, although the interaction between circITCH and HULC has not yet been defined [143]. Aberrant activation of the Wnt/β-catenin signaling plays a key role in promoting the cancer stem cell self-renewal, metastasis and chemoresistance in OC [151]. By sponging miR-9, circPLEKHM3 functions as a tumor suppressor to enhance the expression of BRCA1, DNAJB6 and KLF4, which consequently inactivate the PI3K/AKT and Wnt/β-catenin signaling pathways in OC cells [149].

### 7.3. CircRNAs in EC

To date, very few reports on circRNAs in EC have been published [152,153,154,155,156]. The expression profile of circRNAs was found to be altered in EC tissues compared with the adjacent normal tissues [152]. Similarly, another study identified 62,167 circRNAs that were differentially expressed in grade 3 EC tissues when compared with the adjacent normal tissues [153]. A recent study indicated a positive correlation between RNA-binding protein QKI and circRNAs, and a negative correlation of QKI with the activity of specific miRNAs, suggesting a potential model where QKI, circRNAs, and miRNAs form a regulatory feedback loop in EC [154]. Another study identified differentially expressed circRNAs (including 209 upregulated circRNAs and 66 downregulated circRNAs) in extracellular vesicles isolated from the serum of EC patients compared with healthy controls [155]. CircPUM1 was significantly overexpressed in EC tissues and EC cell lines, and it promoted the development of EC by regulating the miR-136/NOTCH3 axis [156].

## 8. CircRNAs as Potential Biomarkers in Gynecological Cancers

Early studies have provided evidence that circRNAs could be used as biomarkers in distinguishing cancer tissues from normal tissues and predicting the progression and prognosis, and assessment of response to therapy in many cancer types [15]. Dysregulation of circRNAs has been reported to be closely related to the clinicopathological characteristics of patients with CC, OC and EC [79,80,94,117,149,153]. In addition, levels of some circRNAs have been associated with survival times in patients with CC and OC [78,80,141,142,157,158,159,160]. In an in silico analysis of an OC dataset obtained from the GEO database, the expression of six-circRNAs was found to be an independent predictor of overall survival in patients with OC [160].

Moreover, circRNAs can be released into the extracellular space, and subsequently detected in body fluids (such as blood, plasma, serum and exosomes) from patients with gynecological cancers [117,119,137,161,162,163]. Higher serum levels of circSETDB1 have been found in patients with OC compared to healthy controls [161]. Serum levels of circSETDB1 were positively correlated with chemoresistance and shorter survival in patients with OC [161]. CircBNC2 was downregulated in the plasma of patients with OC compared to healthy individuals, and plasma levels of circBNC2 provided a potential tool to distinguish patients with early-stage OC from healthy individuals [164].

The circRNAs that might serve as potential prognostic biomarkers in gynecological cancers are summarized in Table 3.

## 9. CircRNAs as Potential Therapeutic Targets for Gynecological Cancers

Recent data have proved that several circRNAs can influence the growth, survival, invasion, EMT, angiogenesis, and drug resistance in gynecological cancers. Manipulation of circRNA abundance has been shown to possess anti-tumor activities in experimental xenograft models of CC [88,91,95], OC [121,123,124] and EC [156].

The introduction of circRNAs with binding sites for oncogenic miRNAs (or proteins) might sequester miRNAs (or proteins) from native targets, and consequently inhibit the biological activities of tumor-promoting miRNAs (or oncoproteins) in tumor cells [15]. Generally, numerous oncogenic miRNAs are overexpressed in a given tumor, thus artificial circRNAs that contain multiple binding sites for multiple oncogenic miRNAs would be expected to simultaneously bind and inhibit the functions of different tumor-causing miRNAs. Alternatively, circRNAs might be used as stable carriers that deliver tumor-suppressive miRNAs (or proteins) to tumor cells.

In addition, exosomal circRNAs could modulate the progression of cancer, the remodeling of tumor microenvironment, anti-tumor immune response, and the occurrence of metastasis [165]. The discovery that mRNAs from tumor cells can be transfected into dendritic cells and further translated into proteins to function as antigens are intriguing [166,167]. Similar to mRNA, circRNA can be detected in exosomes. Thus, the circRNAs with protein-coding potential might function as potential antigens and be used as potent targets for cancer immunotherapy [168]. Collectively, the research indicates the potential use of circRNAs as therapeutic targets for the treatment of gynecological cancers.

## 10. Conclusions

Despite many exciting advances in the circRNA research area, there are still arguments regarding the functionality of circRNAs [169]. At least, some individual circRNAs have biologically relevant functions in ovarian, cervical and endometrial cancer cells [87,137,156]. Their biogenesis, functions, and action mechanisms in gynecological cancer cells need to be fully investigated. Recent studies suggest that circRNAs may fulfill a regulatory role in gynecological cancers by sponging miRNAs, although the possibility of other mechanisms (such as scaffolds for protein complexes) also exists. CircRNAs have recently been recognized as emerging prognostic biomarkers for gynecological cancers. The unique ability of circRNAs to sponge RNAs (or proteins) and code for proteins make them valuable as potential therapeutic tools to treat gynecological cancers.

## Figures and Tables

**Table 1:** The expression and mechanisms of circRNAs in cervical cancer (CC).

CircRNA	Samples or Cell Lines	Expression	Function	Mechanism	Ref.
circ0018289	CC and adjacent normal tissues; HeLa and SiHa cells	Up	Oncogene	Sponges miR-497	[76]
circ000284	HeLa and SiHa cells	Up	Oncogene	Increases Snail-2 expression via sponging miR-506	[77]
circ0023404	CC and adjacent normal tissues; HeLa and SiHa cells	Up	Oncogene	Activates YAP pathway via promoting TFCP2 expression by sponging miR-136	[78]
circ8924	CC and adjacent normal tissues; HeLa and SiHa cells	Up	Oncogene	Increases CBX8 expression via sponging miR-519a-5p	[79]
circ0067934	CC and adjacent normal tissues; HeLa and SiHa cells	Up	Oncogene	Increases EIF3C expression via sponging miR-545	[80]
circ0000263	HeLa and C33A cells	Up	Oncogene	Inhibits p53 levels by upregulating MDM4 expression via sponging miR-150-5p	[81]
circSMARCA5	CC and adjacent normal tissues; HeLa and C33A cells	Down	TS	Sponges miR-620	[82]
circ101996	CC and adjacent normal tissues; SiHa and CaSki cells	Up	Oncogene	Increases TPX2 expression by sponging miR-8075	[83]
circEIF4G2	CC and adjacent normal tissues; HeLa and C33A cells	Up	Oncogene	Increases HOXA1 expression by sponging miR-218	[84]
circATP8A2	CC and adjacent normal tissues; HeLa and SW756 cells	Up	Oncogene	Increases EGFR expression by sponging miR-433	[85]
circ0023404	CC and adjacent normal tissues; HeLa and SiHa cells	Up	Oncogene	Increases VEGF-A expression by sponging miR-5047	[86]
circE7	CaSki cells	-	Oncogene	Codes HPV E7 oncoprotein	[87]
circMTO1	HeLa and SiHa cells	Up	Oncogene	Increase S100A1 expression by sponging miR-6983	[88]
circ0000745	CC and adjacent normal tissues; CaSki and SiHa cells	Up	Oncogene	Inhibits E-cadherin expression	[89]
circ0007534	CC and adjacent normal tissues; HeLa and SiHa cells	Up	Oncogene	Increases BMI-1 expression by sponging miR-498	[90]
circ0005576	CC and normal cervical tissues; HeLa and SiHa cells	Up	Oncogene	Increases KIF20A expression by sponging miR-153	[91]
circ0000285	CC and adjacent normal tissues; C33A cells	Up	Oncogene	Increases FUS expression	[92]
circAGFG1	HeLa and SiHa cells	Up	Oncogene	Activates MEK/ERK pathway via promoting RAF1 expression by sponging miR-370-3p	[93]
circ0075341	CC and normal cervical tissues; CaSki and SiHa cells	Up	Oncogene	Increases AURKA expression by sponging miR-149-5p	[94]
circ0000515	CC and adjacent normal tissues; HeLa and SiHa cells	Up	Oncogene	Increases ELK1 expression by sponging miR-326	[95]
circ0001038	CC and adjacent normal tissues; HeLa and SW756 cells	Up	Oncogene	Increases Cyclin-M3/MACC1 expression by sponging miR-337-3p	[96]
circAMOTL1	CC, adjacent normal tissues and normal cervical tissues; CaSki and HeLa cells	Up	Oncogene	Increases AMOTL1 expression by sponging miR-485-5p	[97]
circMYBL2	CC, and adjacent normal tissues; CaSki and HeLa cells	Up	Oncogene	Sponges miR-361-3p	[98]
circCLK3	CC and adjacent normal tissues; HeLa and SiHa cells	Up	Oncogene	Induces EMT via promoting FoxM1 expression by sponging miR-320a	[99]
ciRS-7	CC, cervical intraepithelial neoplasia and normal cervical tissues, HeLa, CaSki, C33A and SiHa cells	Up	Oncogene	Increases N-cadherin/Vimentin expression and decreases E-cadherin expression	[100]
circSLC26A4	CC and adjacent normal tissues; CaSki and HeLa cells	Up	Oncogene	Increases HOXA7 expression by sponging miR-1287-5p	[101]
circSMARCA5	CC and adjacent normal tissues; CaSki and HeLa cells	Up	Oncogene	Increases EGFR expression by sponging miR-432	[102]
circITCH	CC and adjacent normal tissues; HeLa cells	Down	TS	Increases FOXK2 expression by sponging miR-93-5p	[103]
circHIPK3	CC and adjacent normal tissues; HeLa and C4I cells	Up	Oncogene	Increases HIF-1α expression by sponging miR-338-3p	[104]
circCSPP1	CC and adjacent normal tissues; HeLa cells	Up	Oncogene	Increases Integrin β1 expression and activate the PI3K/AKT pathway via sponging miR-361-5p	[105]

TS: tumor suppressor; up: upregulation; down: downregulation.

**Table 2:** The expression and mechanisms of circRNAs in ovarian cancer (OC).

CircRNA	Samples or Cell Lines	Expression	Function	Mechanism	Ref.
circEPSTI1	OC and adjacent normal tissues; OV119 and A2780 cells	Up	Oncogene	Increases EPSTI1 expression by sponging miR-942	[121]
circGFRA1	OC and adjacent normal tissues; OV119 and A2780 cells	Up	Oncogene	Increases GFRA1 expression by sponging miR-449a	[122]
circ0051240	OC and adjacent normal tissues; OVCAR-3 and H8910 cells	Up	Oncogene	Increases KLK4 expression by sponging miR-637	[123]
circPIP5K1A	OC and adjacent normal tissues; SKOV-3 and A2780 cells	Up	Oncogene	Increases IGFBP5 expression by sponging miR-661	[124]
circPVT1	SKOV-3 and CAOV3 cells	Up	Oncogene	Sponges miR-149	[125]
circABCB10	OC and adjacent normal tissues; SKOV-3 cells	Up	Oncogene	Inhibits miR-1271, miR-1252 and miR-203 expression	[126]
circSMAD7	OC and adjacent normal tissues; TOV112D cells	Up	Oncogene	Inhibits KLF6 expression	[127]
circANKRD12	SKOV-3 cells	Up	Oncogene	Increases Cyclin-D1 expression	[128]
circCSPP1	OC, borderline tumor and normal ovarian tissues; OVCAR-3 and A2780 cells	Up	Oncogene	Sponges miR-1236-3p	[129]
circ0061140	SKOV-3 and A2780 cells	Up	Oncogene	Promotes EMT by increasing FOXM1 expression via sponging miR-370	[130]
circUBAP2	OC and adjacent normal tissues; OVCAR-3 and H8910 cells	Up	Oncogene	Increases CDH2 expression by sponging miR-144	[131]
circWHSC1	OC and normal ovarian tissues; OVCAR-3 and CAOV3 cells	Up	Oncogene	Increases MUC1/hTERT expression by sponging miR-145/1182	[132,133,134]
circVPS13C- has-circ-001567	OC and adjacent normal ovarian tissues; SKOV-3 and OV-1063 cells	Up	Oncogene	Increases N-cadherin expression and decreases E-cadherin expression	[135]
circCELSR1	Drug-resistant/sensitive OC tissues; SKOV3 and HeyA-8 cells	Up	Oncogene	Increases FOXR2 expression by sponging miR-1252	[136]
circPUM1	OC and normal ovarian tissues; OVCAR-3 and A2780 cells	Up	Oncogene	Increases NF-κB/MMP2 expression by sponging miR-615-5p/miR-6753-5p	[137]
circRhoc	OC and normal ovarian tissues; CAOV3 and A2780 cells	Up	Oncogene	Increases VEGF-A expression by sponging miR-302e	[138,139,140]
circITCH	OC and adjacent normal ovarian tissues; CAOV3 and SKOV-3 cells	Down	TS	Increases RASA1 expression via sponging miR-145	[141]
circITCH	SKOV-3 cells	Down	TS	Sponges miR-10a-α	[142]
circITCH	OC and normal ovarian tissues; UWB1.289 and UWB1.289 + BRCA1 cells	Down	TS	Inhibits lncRNA HULC expression	[143]
circITCH	OC and adjacent normal ovarian tissues; SKOV-3 cells	Down	TS	-	[144]
circCDR1as	OC and normal ovarian tissues; H8910 and A2780 cells	Down	TS	Increases HIF1AN expression by sponging miR-135b-5p	[145]
circCDR1as	Drug-resistant/sensitive OC tissues; SKOV3 and A2780 cells	Down	TS	Increases SCAI expression by sponging miR-1270	[146]
circ0007874	SKOV3 and A2780 cells	Down	TS	Increases SOCS3 expression by sponging miR-760	[147]
circLARP4	SKOV3 and A2780 cells	Down	TS	Increases LARP4 expression by sponging miR-513b-5p	[148]
circPLEKHM3	Primary OC, metastatic OC and normal ovarian tissues; A2780 and MDAH2274 cells	Down	TS	Inactivates the PI3K/AKT and Wnt/β-catenin pathways via promoting BRCA1, DNAJB6a and KLF4 expression by sponging miR-9	[149]
circ100395	OC and adjacent normal ovarian tissues; SKOV-3 and ES-2 cells	Down	TS	Increases E-cadherin expression and reduces N-cadherin and Snail expression via promoting p53 expression by sponging miR-1228	[150]

TS: tumor suppressor; up: upregulation; down: downregulation.

**Table 3:** CircRNAs as potential biomarkers in gynecological cancers.

circRNA	Cancer	Expression	Sample	Clinicopathologic Features	Prognosis	Ref.
circ0023404	CC	Up	Tissue	-	OS	[78]
circ8924	CC	Up	Tissue	Size, stage, tumor invasion	-	[79]
circ0067934	CC	Up	Tissue	Stage, LNM	OS	[80]
circ101996	CC	Up	Tissue	Stage, LNM, size	OS	[83]
circEIF4G2	CC	Up	Tissue	Size, LNM	OS	[84]
circATP8A2	CC	Up	Tissue	Stage, LNM, tumor invasion	OS	[85]
circ0000745	CC	Up	Tissue	Grade, vascular/lymphatic invasion	-	[89]
circ0005576	CC	Up	Tissue	Stage, LNM	OS	[91]
circ0075341	CC	Up	Tissue	Size, stage, LNM	-	[94]
circ0000515	CC	Up	Tissue	-	OS	[95]
circ0001038	CC	Up	Tissue	LNM, tumor invasion	OS	[96]
circAMOTL1	CC	Up	Tissue	Tumor metastasis, stage	OS	[97]
circMYBL2	CC	Up	Tissue	Stage, size, LNM	-	[98]
circCLK3	CC	Up	Tissue	Grade, stage, tumor invasion	OS, DFS	[99]
ciRS-7	CC	Up	Tissue	Size, stage, tumor invasion, LNM, HPV infection	-	[100]
circSLC26A4	CC	Up	Tissue	-	OS	[101]
circ1656	OC	Down	Tissue	Stage	-	[117]
circ0051240	OC	Up	Tissue	Tumor metastasis, LNM, stage	OS	[123]
circPIP5K1A	OC	Up	Tissue	-	OS	[124]
circABCB10	OC	Up	Tissue	Grade, size, stage	OS	[126]
CircCSPP1	OC	Up	Tissue	Stage, grade	-	[129]
circUBAP2	OC	Up	Tissue	Stage	OS	[131]
circWHSC1	OC	Up	Tissue	Grade	-	[132]
circVPS13C-has-circ-001567	OC	Up	Tissue	Stage, LNM	-	[135]
circCELSR1	OC	Up	Tissue	Chemoresistance	-	[136]
circPUM1	OC	Up	Tissue	Stage	-	[137]
circRhoC	OC	Up	Tissue	Stage, grade	-	[140]
circITCH	OC	Down	Tissue	-	OS	[141]
circITCH	OC	Down	Tissue	Size, stage, grade	OS	[142]
circCDR1as	OC	Down	Tissue	chemoresistance	-	[145]
circPLEKHM3	OC	Down	Tissue	Tumor metastasis	OS, DFS	[149]
circ100395	OC	Down	Tissue	LNM, tumor metastasis, stage	OS	[150]
circ0039569	EC	Up	Tissue	Grade	-	[153]
circLARP4	OC	Down	Tissue	Stage, LNM	OS, DFS	[157]
circHIPK3	OC	Up	Tissue	LNM, stage	OS, DFS	[158]
circEXOC6B	OC	Down	Tissue	Age, LNM	OS	[159]
circN4BP2L2	OC	Down	Tissue	Age, stage, LNM	PFS	[159]
circSETDB1	OC	Up	Serum	Stage, LNM, chemoresistance	PFS	[161]
circBNC2	OC	Down	Plasma	Grade, histological subtype, LNM, tumor metastasis	-	[164]

Up: upregulation; down: downregulation; CC: cervical cancer; EC: endometrial cancer; OC: ovarian cancer; LNM: lymph node metastasis; OS: overall survival; DFS: disease-free survival; PFS: progress-free survival.
